# Dried plum diet protects from bone loss caused by ionizing radiation

**DOI:** 10.1038/srep21343

**Published:** 2016-02-11

**Authors:** A.-S. Schreurs, Y. Shirazi-Fard, M. Shahnazari, J. S. Alwood, T. A. Truong, C. G. T. Tahimic, C. L. Limoli, N. D. Turner, B. Halloran, R. K. Globus

**Affiliations:** 1 Bone and Signaling Laboratory, Space Biosciences Division, NASA Ames Research Center; 2 Department of Radiation Oncology, University of California Irvine; 3 Department of Nutrition and Food Science, Texas A&M University; 4 Department of Medicine, Division of Endocrinology, University of California San Francisco.

## Abstract

Bone loss caused by ionizing radiation is a potential health concern for radiotherapy patients, radiation workers and astronauts. In animal studies, exposure to ionizing radiation increases oxidative damage in skeletal tissues, and results in an imbalance in bone remodeling initiated by increased bone-resorbing osteoclasts. Therefore, we evaluated various candidate interventions with antioxidant or anti-inflammatory activities (antioxidant cocktail, dihydrolipoic acid, ibuprofen, dried plum) both for their ability to blunt the expression of resorption-related genes in marrow cells after irradiation with either gamma rays (photons, 2 Gy) or simulated space radiation (protons and heavy ions, 1 Gy) and to prevent bone loss. Dried plum was most effective in reducing the expression of genes related to bone resorption (*Nfe2l2, Rankl, Mcp1, Opg, TNF-α*) and also preventing later cancellous bone decrements caused by irradiation with either photons or heavy ions. Thus, dietary supplementation with DP may prevent the skeletal effects of radiation exposures either in space or on Earth.

Radiation exposure of skeletal tissues can lead to bone loss and therefore is relevant to astronauts^1^,^2^,^3^,^4^,^5^,^6^,^7^,^8^,^9^, cancer patients undergoing radiotherapy^10^,^11^,^12^,^13^, radiation workers and victims of nuclear accidents^14^,^15^,^16^. Reduced bone density (osteopenia) and osteoporosis, are major health conditions affecting millions of people across the world, and osteoporotic patients are increasing with an aging population^17^. Extensive research focuses on both understanding the fundamental underlying mechanisms and finding suitable treatments for such patients. However, major health concerns remain, such as secondary drug effects, osteonecrosis of the jaw and atypical femoral fractures^18^,^19^,^20^,^21^, motivating continued search for osteoprotective treatments against bone loss.

Space radiation is a unique type of radiation, composed of various ions and photons due to the combination of galactic cosmic rays (GCR), solar particle events (SPE), and protons and electrons trapped in the magnetosphere^22^,^23^. Linear energy transfer (LET) of a given ion species reflects its ionization capacity and the heavier the nuclei (High-Z High-Energy, HZE), the more primary and secondary ionizations occur for each particle traversal^22^,^23^. Galactic space radiation is composed of mostly low-LET species including proton (^1^H, 87%) and helium ions (alpha-particles, 12%) but also HZE particles, including iron (^56^Fe)^22^. High-LET radiation increases the multiplicity of damage types on a variety of target molecules in the cell, and poses significant challenges to cellular repair processes when this complex damage occurs on the DNA^23^.

In contrast to radiation doses experienced on the International Space Station (ISS) (range: 0.4 to 1.1 mGy/day)^23^, future long duration missions outside the protection of the Earth’s magnetosphere, or unshielded exposures to SPE, may achieve total doses capable of causing cancellous bone loss (i.e. 0.5–2 Gy)^22^,^24^. It is estimated that the maximum dose of exposure to space radiation over a protracted 3-year mission outside the magnetosphere would be ~2 Gy^22^,^24^, which equals the typical single fraction dose delivered during standard clinical radiotherapy^13^,^21^.

Cancellous bone loss caused by ionizing radiation occurs quite rapidly in rodents (within two weeks). Initially, radiation increases the expression of pro-osteoclastogenic cytokines in both marrow cells and mineralized tissue and increases the number and activity of bone-resorbing osteoclasts^5^,^25^,^26^,^27^,^28^. Total body irradiation (TBI) increases the generation of reactive oxygen species (ROS) by marrow cells^1^ and stimulates gene expression levels of pro-osteoclastogenic cytokines such as *Rankl*, *Mcp1*, *Tnf-α* and *Il-6* in addition to *Nfe2l2*^29^. *Nfe2l2* encodes a master transcription factor that regulates the expression of more than 600 genes to help defend the cell from damaging ROS and resulting oxidative stress^30^. We showed previously that increases in gene expression for *Nfe2l2* and osteoclastogenic cytokines one day after TBI precede the manifestation of bone loss (11 days post-TBI)^29^.

The changes in remodeling activity caused by exposure to radiation can lead to impaired structural integrity and fragility both in animal models and radiotherapy patients^3^,^13^,^21^. Radiation-induced bone loss resembles accelerated, age-related structural changes, particularly in rapid-turnover cancellous tissue. Over the course of three days to one month, relatively low doses of radiation (<2 Gy) can cause rapid and progressive strut thinning and removal of cancellous tissue as shown by our group and others^3^,^5^,^25^,^26^,^27^,^31^.

We hypothesized that diets or drugs capable of preventing the early increase in pro-osteoclastogenic and oxidative stress-related factors mitigate cancellous bone loss caused by both low LET and high LET radiation. To test this, we evaluated the following candidate interventions: (#1) an antioxidant diet cocktail (AOX), including 5 antioxidants (ascorbic acid, N-acetyl cysteine, L-selenomethionine, dihydrolipoic acid and vitamin E) reported to protect target tissues from ionizing radiation^32^,^33^,^34^, (#2) dihydrolipoic acid (DHLA), which possesses antioxidant properties^4^,^35^, (#3) Ibuprofen, an anti-inflammatory drug, to test the ability to prevent inflammation-related bone loss^36^,^37^ and (#4) Dried Plum (DP, 25% by weight), shown to mitigate age-related bone loss as an anti-resorptive in other disease models^38^,^39^,^40^,^41^,^42^.

The candidate interventions first were evaluated using early gene expression markers (pro-osteoclastogenic cytokines, inflammatory and antioxidant responses)^29^ one day after TBI. Interventions were then tested for their ability to prevent radiation-induced cancellous bone loss. Thus, analysis of early changes in gene expression levels for osteoclastogenic cytokines after exposure to ionizing radiation served both to identify potentially effective interventions, and to further establish the relevance of early changes in marrow cytokine expression for cancellous bone loss. Here we report that one of our selected interventions (DP diet) completely prevented cancellous bone loss caused by ionizing radiation.

## Results

### Food consumption and body weights

**Figure 1:**
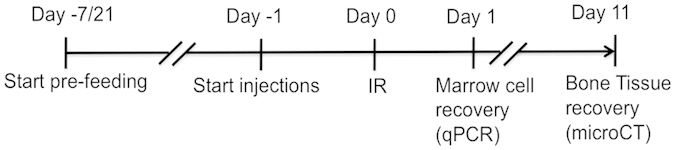
Experiment design. Male mice were assigned to groups (n = 5–10/group) and pre-fed for 7 to 21 days with the various diets (Control diets, CD; or customized diet, AOX and DP), or injected twice a day with DHLA or Ibuprofen starting one day prior to TBI and until tissue harvest. Mice were exposed at 16 wk of age to TBI with 2 Gy Gamma or 1 Gy of dual protons and ^56^Fe. Tissues were harvested 24 hours later for gene expression or 11 days later for microCT analysis.

To evaluate overall health, body weights and food consumption were monitored throughout the experiments (Table 1). Candidate interventions were administrated to the mice prior to TBI using pre-feeding protocols reported to effectively protect other tissues^34^,^43^,^44^,^45^,^46^ or bone^47^ and following exposure to radiation as described in the methods (Fig. 1). There were no significant differences in food consumption or final body weights within diet groups due to irradiation (Table 1), indicating that the various diets were well tolerated. Hence, difference in body weights was not a factor for the differences observed in skeletal properties.

### Mechanisms of radiation-induced bone loss and assessment of interventions

**Figure 2:**
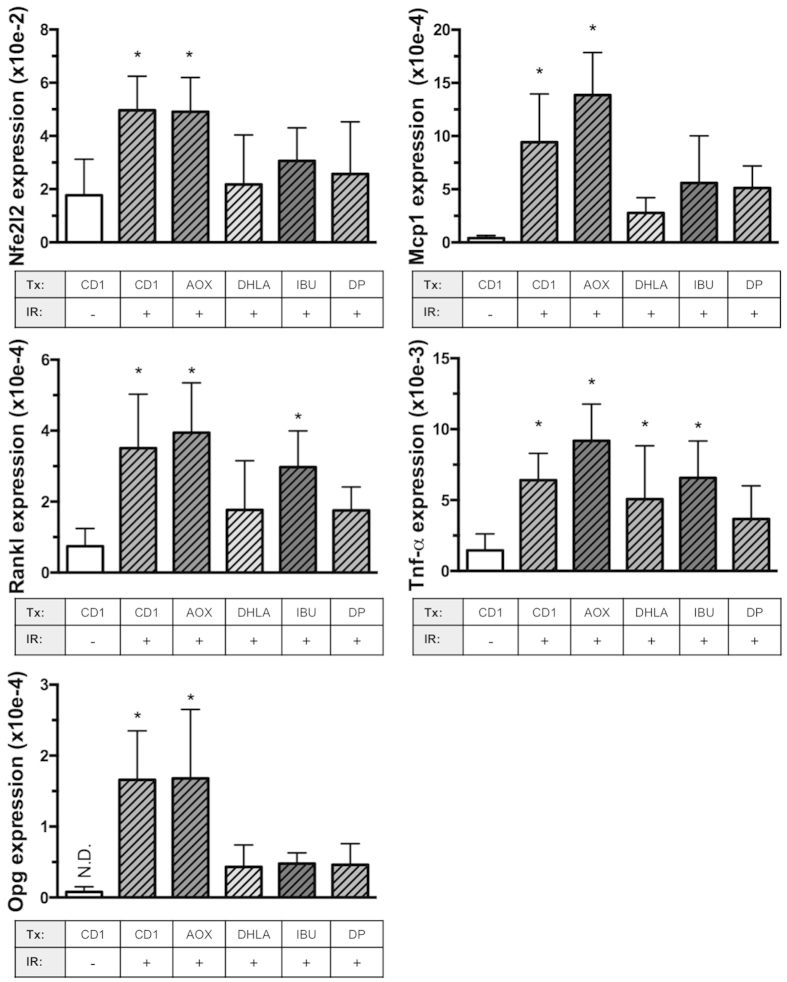
Effects of candidate interventions on radiation-induced increase of resorption-related genes. Mice were fed various diets or injected with DHLA or Ibuprofen, then irradiated with ^137^Cs (2 Gy). Dietary interventions included an antioxidant cocktail (AOX) or dried DP (25% by weight) and three separate control diets (Fig. 1 and Methods). After irradiation (24 hr + / − 20 min), mice were euthanized and bone marrow cells were collected for analysis of gene expression by qPCR. Y-axis values indicate relative expression levels of gene of interest normalized to *Gapdh* using the ΔCt method. Data shown are mean + S.D. (n = 5–6/group) and analyzed by 1-factor ANOVA. *indicates p < 0.05 compared to CD1/sham-irradiated controls by Dunnett’s post hoc test.

To test candidate interventions for their ability to mitigate elevated expression levels of pro-osteoclastogenic and antioxidant genes caused by irradiation, bone marrow was recovered 24 hours after exposure then analyzed by qPCR (Fig. 2). In the control diet (CD)-fed groups, radiation exposure led to an increase in marrow cell expression of genes associated with bone resorption, including the osteoclastogenic cytokine *Rankl* (1.5-fold increase), monocyte chemokine attractant *Mcp1* (6-fold increase), and the pro-inflammatory molecule *Tnf*-α (5-fold increase). Opg, the decoy receptor for RANKL, was not within range of detection in control, sham-irradiated animals, but exhibited increased expression levels after irradiation. In addition, radiation caused a two-fold increase in expression levels of the global antioxidant transcription factor, *Nfe2l2*. These changes in pro-osteoclastogenic cytokine and antioxidant gene expressions were consistent with our previous findings^29^.

DP was effective in maintaining all gene expression levels comparable to controls (i.e. control diet, sham-irradiated) one day after exposure to gamma irradiation (Fig. 2). The inhibitory effects of DHLA treatment were comparable to DP except for the apparent suboptimal effect of DHLA to inhibit *Tnf*-α expression. Ibuprofen only mitigated the expression of *Nfe2l2, Mcp1 and Opg.* Surprisingly, the AOX diet did not prevent the changes in gene expression caused by radiation, although it effectively counteracts other types of radiation damage^32^,^33^,^34^.

### DP effects on bone loss caused by gamma irradiation

**Figure 3:**
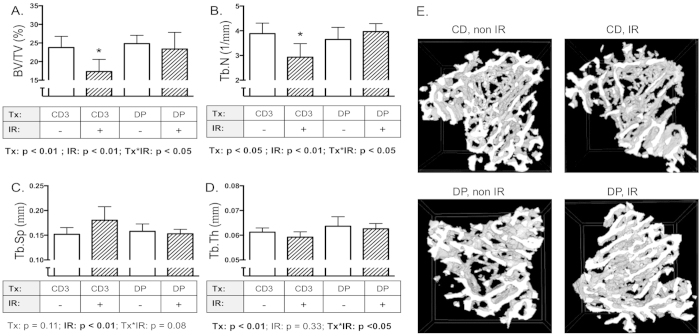
DP diet prevented gamma radiation-induced cancellous bone loss. Mice were fed control diet (CD3) or DP diet, then irradiated with ^137^Cs (2 Gy). After irradiation (11d), mice were euthanized and tibiae collected. Bones were analyzed by microCT for percent bone volume (BV/TV, Panel **A**), trabecular number (Tb.N, Panel **B**), trabecular separation (Tb.Sp, Panel **C**) and trabecular thickness (Tb.Th, Panel **D**). Representative images of the cancellous bone microarchitecure in 3D reconstructions using the microCT are shown in (panel **E**). Data shown are mean + S.D. (n = 8/group) and analyzed by 2-factor ANOVA. *indicates p < 0.05 compared to CD3/sham-irradiated controls by Dunnett’s post hoc test.

Since DP exhibited the most definitive protection from radiation-induced increases in expression levels of the pro-resorption genes tested in this study, we then assessed its ability to prevent associated decrements in skeletal microarchitecture. Mice were fed DP for 17 days (Fig. 1), then exposed to 2 Gy gamma radiation and tissues were recovered 11 days later, a regimen well established to induce cancellous bone loss^5^,^26^. Microcomputed tomography (microCT) measurements were analyzed by 2-factor ANOVA and revealed main effects in percent Bone Volume/Total Volume (BV/TV) for radiation and diet as well as interaction effects (Fig. 3A). Radiation caused a 32% decrement in BV/TV (Fig. 3A), a 25% decrease in trabecular number (Tb.N, Fig. 3B), and a 13% increase in trabecular separation (Tb.Sp, Fig. 3C) compared to sham-irradiated controls fed the control diet (CD3). Trabecular thickness (Tb.Th, Fig. 3D) was unaffected by irradiation, consistent with our prior findings^26^,^48^. In contrast, mice on the DP diet did not exhibit decrements after exposure to 2 Gy ^137^Cs in any of the structural parameters, indicating potent radioprotective effects of DP against cancellous bone loss.

The tibial shaft was analyzed by microcomputed tomography to determine if DP diet or irradiation affected structural (perimeter, thickness, area, moment of inertia) or material properties (tissue mineral density) of cortical tissue, which mainly contribute to whole bone mechanical properties (defining bone strength). Consistent with previous findings, irradiation did not affect cortical bone structure (Table 2), nor did feeding the DP diet.

**Figure 4:**
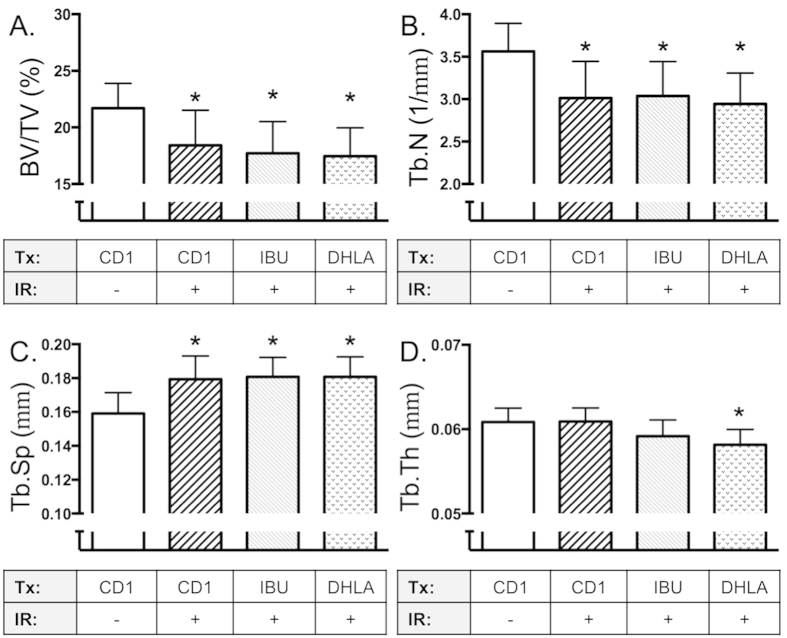
Effects of DHLA and Ibuprofen on radiation-induced bone loss. Mice were injected with DHLA, Ibuprofen or Vehicle as described in the methods, then irradiated with ^137^Cs (2 Gy). After irradiation (11d), mice were euthanized and tibiae collected and analyzed by microCT. (BV/TV, Panel **A**), Tb.N (Panel **B**), Tb.Sp (Panel **C**) and Tb.Th (Panel **D**). Data shown are mean + S.D. (n = 8/group) and analyzed by 1-factor ANOVA. *indicates p < 0.05 compared to CD1/sham-irradiated controls by Dunnett’s post hoc test.

In contrast to the DP diet-supplemented groups, Ibuprofen and DHLA-treated mice displayed decrements in cancellous microarchitecture similar to irradiated animals that were not provided any treatment (Fig. 4A–D).

### Effects of interventions on cancellous bone structure after exposure to simulated space radiation

**Figure 5:**
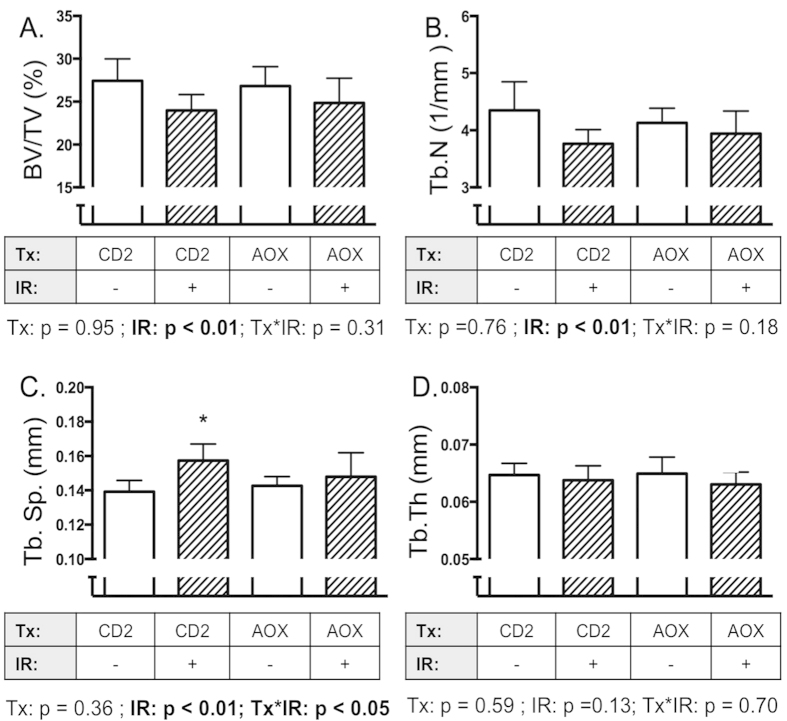
Effects of AOX diet on cancellous bone loss induced by simulated spaceflight radiation. Mice were fed control diet (CD2) or AOX diet, then irradiated with dual ions (protons, ^56^Fe) to simulate space radiation. After irradiation (11d), mice were euthanized and tibiae collected then analyzed by microCT. (BV/TV, Panel **A**), Tb.N (Panel **B**), Tb.Sp (Panel **C**) and Tb.Th (Panel **D**). Data shown are mean + S.D. (n = 10). *indicates p < 0.05 compared to CD2, 0 Gy.

Because HZE and protons, comprising the major species of space radiation, exert effects that can differ from gamma radiation, we examined the effectiveness of candidate antioxidant treatments (AOX diet, DHLA and DP) after TBI with simulated space radiation (Fig. 1, Methods). AOX also was included to test a corollary hypothesis that an unchecked increase in pro-osteoclastogenic gene expression leads to bone loss in a space radiation model. As expected, irradiated animals on the control diet showed decrements in percent bone volume and other structural parameters compared to sham-treated animals on the same diet. Irradiation with simulated space radiation caused a main effect of radiation but not diet, on BV/TV (Fig. 5A). Consistent with the corollary hypothesis, AOX diet did not prevent the radiation-induced decreases in BV/TV and Tb.N (Fig. 5A,B), but appeared to exert a modest protective effect on Tb.Sp (Fig. 5C). No effects were observed in Tb.Th as expected (Fig. 5D).

**Figure 6:**
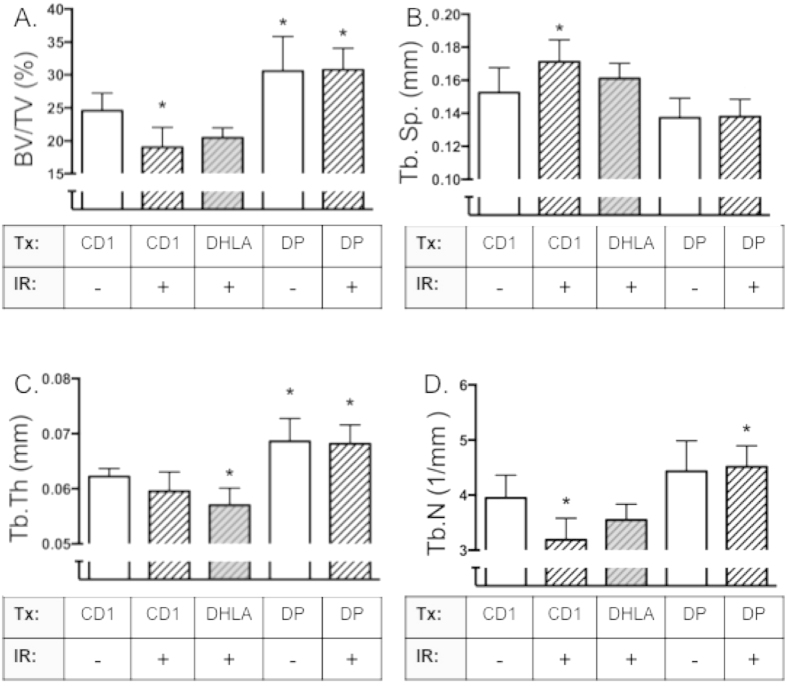
DP, but not DHLA, completely prevented bone loss induced by simulated spaceflight radiation. Mice were separated into groups (n = 8/group), fed selected diets (CD1 or DP) or were injected with DHLA, then were irradiated with dual ions (protons, ^56^Fe) to simulate space radiation. Mice were euthanized 11 days post-IR, and bones analyzed by microCT for percent bone (BV/TV, Panel **A**), Tb.N (Panel **B**), Tb.Sp (Panel **C**) and Tb.Th (Panel **D**). Data shown are mean + S.D. (n = 8). *indicates p < 0.05 compared to CD1 0 Gy.

DHLA did not prevent radiation-induced bone loss (Fig. 6A–D), similar to the results obtained with gamma-irradiation. In contrast, DP fully preserved cancellous percent bone volume and other structural parameters after irradiation, suggesting its potential as a radiomitigator for HZE and proton exposures (Fig. 6A–D).

## Discussion

Exposure to ionizing radiation caused both a rapid increase in expression of pro-osteoclastogenic cytokines (day 1 post-IR) and a later decrement in cancellous BV/TV and microarchitectural integrity (day 11 post-IR), consistent with our previous findings^29^. DP was effective at reducing expression of early pro-osteoclastogenic cytokines, and an important indicator of antioxidant responses, *Nfe2l2*, in bone marrow. DP also completely prevented microarchitectural deficits, whereas other treatments (DHLA, IBU) did not. The AOX diet, which effectively mitigates morbidity caused by exposure to high doses radiation (8 Gy)^32^,^33^, failed to prevent effects of radiation on expression of osteoclast-related genes and subsequent bone loss.

Our study demonstrates the complexity of the processes underlying bone loss caused by exposure to radiation. Results indicate that co-existence of high levels of pro-resorption, pro-inflammation, and oxidative stress-related genes in the bone marrow strongly correlated with cancellous bone loss. Treatments that failed to mitigate the alterations in these molecular markers (AOX and IBU) ultimately were unsuccessful in mitigating radiation-induced decrements in skeletal microarchitecture. This suggests that preventing up-regulation of these molecular responses should be considered in the development of a rational strategy to mitigate bone loss. However, seemingly paradoxical is the observation that DHLA, which appeared nearly as effective as DP in preventing radiation-induced increases in expression of these markers, did not protect skeletal integrity. A plausible explanation for this observation is that there are other equally important determinants of bone loss apart from up-regulation of these molecular markers. In this case, DP was clearly more effective at ameliorating most of these determinants than DHLA as DP abrogated the decrements in BV/TV caused by radiation. In addition, AOX and DP diets displayed similar total antioxidant capacity, suggesting that antioxidant capacity of the diets alone, as measured by this assay, is not sufficient to protect bone from radiation. This again, is consistent with the idea that the determinants of bone loss are multi-factorial.

DP is known to inhibit resorption in models of aging and ovariectomy-induced osteopenia^40^,^49^ as do other polyphenol-rich fruits (e.g. blueberries^50^,^51^), although prior to this study, radioprotective effects of dried plum were not reported. The mechanism mediating DP radioprotection is uncertain, although there is evidence that specific components including polyphenols, promote osteogenesis and prevent osteoclastogenesis^39^,^52^. Purified dried plum polyphenols (DPP) contains various polyphenols such as gallic acid, caffeoyl-quinic acids, coumaric acid and rutin. These polyphenols are known for their high antioxidant and anti-inflammatory properties. Consistent with these *in vitro* findings, DP diet prevented IR-induced elevation in levels of *Nfe2l2* and *Tnf-α in vivo* compared to animals fed with controls diets. In the context of spaceflight relevance, it will be of particular interest to determine the ability of the DP diet to prevent simulated or actual microgravity-induced bone loss as musculoskeletal disuse leads to deficits in both cortical and cancellous compartments of bone^4^,^53^,^54^,^55^.

Several *in vitro* studies demonstrate the potential of DP to prevent free radical damage as well as inflammatory responses in RAW 264.7 cells (osteoclast-like cell line) and MC3T31 cells (osteoblast-like cell line)^52^,^56^,^57^. In the context of skeletal remodeling, purified polyphenols from DP powder inhibit bone-resorbing osteoclastogenesis *in vitro* by down-regulating osteoclast differentiation and expression of osteoclast-specific genes (*Rankl*, *Nfatc1*) in RAW264.7 cells after treatment with lipopolysaccharide or H_2_O_2_^52^. In addition, DPP enhances differentiation of an osteoblast cell line (MC3T3E1) *in vitro* both under normal conditions, as well as after treatment with TNF-*α*^57^. Together these findings indicate that DP polyphenols may exert beneficial effects on remodeling by inhibiting bone resorption and/or improving bone formation.

Beyond the scope of this study but topics worthy of future investigation, include a determination of the active component(s) of plum that exert(s) the observed protective effects. It still has to be linked directly *in vivo* that the dried plum polyphenols are the active compound exerting the radio-protective effects. In addition, even when purified, DP contains multiple polyphenols, and it remains uncertain whether the majority of the beneficial effects of DP are derived from a single or more complex combination of polyphenols. Moreover, the roles and properties of other bioactive compounds in plum (pectin, polysaccharides, lycopenes, iron-chelators, etc) in bone remodeling have yet to be determined^58^,^59^,^60^. Studies conducted to date using the DP diet suggest that the combination of multiple constituents may be needed to exert full protection against radiation-induced bone loss^58^,^59^,^60^,^61^,^62^. In addition, long duration experiments with DP and ionizing radiation would be valuable to assess whole bone mechanical properties along with structure, as cortical changes develop more slowly due to the lower metabolic activity of cortical tissue compared to cancellous tissue. Nonetheless, the relatively rapid changes (within 11d) in cancellous structure observed due to exposure to ionizing radiation are likely to be biologically relevant as radiation caused the removal of trabecular struts (TbN), which generally is irreversible^3^,^63^,^64^, and DP diet prevented this strut loss.

In summary, DP completely prevented cancellous bone loss caused by irradiation over this short duration study (11day post-IR). Our studies demonstrated that DP diet supplementation was equally effective at preventing the skeletal responses to both low-LET gamma (^137^Cs) radiation, at a dose equivalent to a single fraction of radiotherapy, or combined proton and HZE ions, simulating space radiation. Therefore DP or its components may provide effective interventions for loss of structural integrity caused by radiotherapy or unavoidable exposure to space radiation incurred over long duration spaceflight.

## Methods

### Animals

Male C57BL/6J mice (Jackson Labs, Sacramento, CA for the experiments conducted at Ames Research Center and Bar Harbor, ME for the Brookhaven National Laboratory (BNL) experiments) at 16 weeks of age were randomized by weight, individually housed, and assigned to groups (n = 5–10/group). Food and water were made available *ad libitum* and mice were housed on a 12 hours light/dark cycle. Body weights and food consumption were measured throughout the experiments to monitor animal health (Table 1). The NASA Ames Research Center and the Brookhaven National Laboratory Institutional Animal Care and Use Committee (IACUC) approved all procedures, and studies were conducted in accordance to the IACUC health and ethical standards.

### Diets

**Figure 7:**
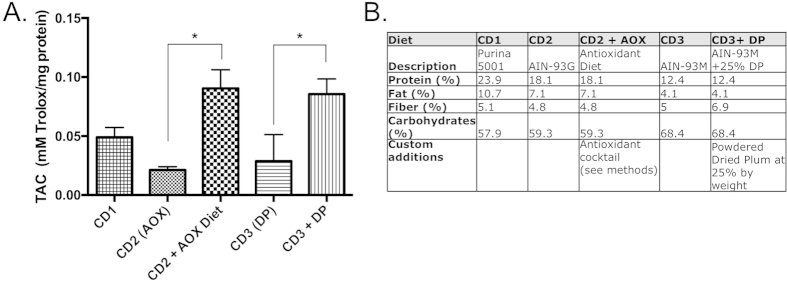
Dietary total antioxidant capacity (TAC) and composition. (Panel **A**) TAC was measured in the various diets to assess relative antioxidant quality of the custom food sources. CD1 (Purina 5001) was the laboratory’s standard diet, CD2 (AIN93G), was the control for the AOX-supplemented diet and CD3 (AIN-93M) was the control for DP (Fig. 1 and methods). Data shown are mean + S.D. from 3–4 separate aliquots. *p < 0.05 compared to their respective controls (Panel **B**). Composition of the diets: Control Diet 1 (CD1) is the LabDiet 5001. The specialized diet CD2 + AOX (i.e. antioxidant cocktail diet) was customized supplementing with 5 antioxidants: ascorbic acid (AA), N-acetyl cysteine (NAC), L-selenomethionine, lipoic acid (DHLA), and vitamin E (See Methods). The customized dried plum (DP) diet is the AIN93M diet with addition of 25% by weight of dried plum powder.

Diet compositions are shown in Fig. 7. The control diets included the following: Control Diet 1 (CD1) was LabDiet 5001; Control Diet 2 (CD2) was purified AIN93G (Bio-Serv, Frenchtown, NJ) and was used as a control for the AOX-supplemented diet of A. Kennedy and colleagues^34^. Control Diet 3 (CD3) was AIN93M (Teklad, Madison, WI) and served as the control for the DP-supplemented diet^40^. The custom antioxidant diet (CD2 + AOX), was prepared by a commercial vendor (Bio-Serv, Frenchtown, NJ) based on a previously reported diet composition^32^,^33^, with the base AIN93G diet supplemented with five antioxidants: ascorbic acid (142.8 mg/kg of diet), N-acetyl cysteine (171.4 mg/kg of diet), L-selenomethionine (0.06, mg/kg of diet), dihydrolipoic acid (DHLA, 85.7 mg/kg of diet), and vitamin E (71.4 mg/kg of diet). All antioxidants were obtained from Sigma (Sigma-Aldrich, St. Louis, MO). The DP diet was composed of 25% by weight powdered dried plum (a gift from the California Dried Plums Board) and was prepared by Teklad as reported by Halloran *et al.*^40^.

### Analysis of custom diets

The candidate interventions were selected in part on the basis of their antioxidant properties. Antioxidant capacity of the custom diets was measured using a Total Antioxidant Capacity (TAC) measurement assay (Oxford Biomedical)^38^. In this assay, levels of Trolox equivalents are positively correlated with antioxidant content. DP and DHLA diets had significantly higher antioxidant capacities (Fig. 7) compared to their respective controls. Specifically, Control diet 1 (CD1, LabDiet 5001) contained 0.05 mM Trolox equivalent per mg of protein. Control diets 2 and 3, CD2 (control diet for the antioxidant diet); and CD3 (control diet for the DP diet) had lower TAC compared to CD1, 56% and 41% respectively. Both the antioxidant cocktail (AOX) and dried DP (DP) diets had 84% and 74% increased TAC compared to CD1, and their respective controls at 323% and 196% TAC. The AOX and DP diets had comparable TAC’s (Fig. 7).

### Feeding and injection protocols

Mice were separated into groups and fed various diets (Fig. 7) or injected with DHLA (25 mg/kg BW twice a day, at 12 hours interval), Ibuprofen (10 mg/kg BW twice a day, at 12 hours interval) (Fig. 1). Mice were pre-fed 7 days (for the AOX diet), 17 days (for the DP diet) or 21 days (for the gene expression analysis) with the diets (Control Diet (CD1), AOX or DP) prior to total body irradiation (TBI). The differences in pre-feeding periods were based on previous published findings showing effective prevention of radiodamage by AOX diet^34^,^43^,^44^,^45^,^46^ or bone loss prevention by DP^47^. Feeding with the corresponding diets was continued until euthanasia. For the injection protocols, DHLA (25 mg/kg body weight) or Ibuprofen (10 mg/kg body weight) was delivered via subcutaneous injection twice a day, starting one day (24 hours) prior to TBI as previously reported^29^. In all experiments, mice were irradiated at 16 weeks of age and tissues harvested 1 day later for the gene expression analysis, and 11 days later for the microCT analysis (Fig. 1).

### Radiation exposure

Conscious mice were exposed (TBI) at 16 weeks of age to 2 Gy Gamma (^137^Cs at 83cGy/min, JL Shepherd Mark I, NASA ARC) or with 1 Gy of protons and ^56^Fe ions delivered sequentially to simulate space radiation. The sequential ion exposure was comprised of an initial exposure to 25cGy of ^1^H (3cGy/min, at 150 MeV/ion), then 50cGy of ^56^Fe (5cGy/min, at 600 MeV/ion), and, finally, 25cGy of ^1^H; for a total dose of 1 Gy, and was performed at the NASA Space Radiation Laboratory (NSRL) at Brookhaven National Laboratory (BNL), NY. This exposure regimen was designed to simulate space radiation combining both low-LET (proton) and high-LET (such as iron) particles. Controls were handled identically to the irradiated animals with the exception of exposure to the radiation source, and are referred to as ‘sham-irradiated’.

### Gene expression

Flushed bone marrow cells from the femora and tibiae were pelleted and lysed in RLT buffer with 1% beta-mercaptoethanol and stored at  −80C. Total RNA was extracted using QIAshredder, and RNeasy mini kit (Qiagen, Inc., Valencia, CA, USA). For each sample, RNA was treated with RNase-free DNase Set (Qiagen, Inc., Valencia, CA, USA). RNA quantity was determined using a spectrophotometer (NanoDrop, Wilmington, DE, USA) and quality confirmed by 2100 Bioanalyzer (Agilent Technologies, Santa Clara, CA, USA). Equal amounts of RNA were reversed transcribed, followed by qPCR using GoTaq^®^ RT-qPCR System (Promega, Madison, WI, USA). Taqman gene expression assays were used (Applied Biosystems, Inc., Foster City, CA, USA): nuclear factor, erythroid 2-like 2 (*Nfe2l2*, assay ID: Mm00477784_m1), receptor activator of nuclear factor kappa-B ligand (*Rankl*, assay ID: Mm00441906_m1), osteoprotegerin (*Opg*, assay ID: Mm01205928_m1), tumor necrosis factor alpha (*Tnf*, assay ID: Mm00443260_g1) and monocyte chemotactic protein-1 (*Mcp1*, assay ID: Mm00441242_m1). The relative gene expression was quantified using the comparative threshold cycle method (DeltaDeltaCt) with normalization to expression levels of glyceraldehyde 3-phosphate dehydrogenase (*Gapdh*, assay ID: Mm99999915_g1). The reactions were performed in a 7300 RT-PCR System (Applied Biosystems, Foster City, CA) as described previously^29^.

### Microcomputed tomography (MicroCT)

Tibiae were dissected, cut distal to the TFJ (Tibia-Fibula Junction) to allow PFA (5% PFA, Sigma) infiltration and fixed for 24 hours at 4 °C, followed by storage in 70% Ethanol. The bones were transferred to phosphate-buffered saline (PBS) and then the proximal metaphysis (i.e., cancellous) scanned and analyzed as previously described^29^,^48^ with a 6.7 μm/voxel resolution using a SkyScan 1174 microCT scanner (3500 ms integration time, 50 kV; Kontich, Belgium). For cancellous bone, a 1.0 mm thick region located 0.24 mm distal to the proximal growth plate of the tibia was selected and semi-autonomously contoured to include cancellous tissue. To assess cancellous bone loss, the bone volume to total volume fraction (BV/TV, %), trabecular thickness (Tb.Th, mm), trabecular number (Tb.N, 1/mm), and trabecular separation (Tb.Sp, mm) were calculated and reported following conventional guidelines^48^.

To measure possible changes in cortical features that contribute to whole bone mechanical properties, bones were scanned at 14.6 μm/voxel resolution starting at midshaft 2 mm proximal to the tibia–fibula junction over a 0.3 mm height. Parameters reported include cortical bone cross-sectional area (bone area, mm^2^), bone periosteal perimeter (bone perimeter, mm), cortical thickness (Ct.Th, mm) and mean polar moment of inertia (mm^4^). Tissue mineral density (TMD, g/cm^3^) was calculated using the linear attenuation coefficient and calibrated phantoms for diaphyseal cortical bone.

## Statistics

A one-way or two-way analysis of variance (ANOVA) was performed as indicated in the legends, with treatment (diet, injection) and irradiation as main effects. Where the main effect P < 0.05 by 1-factor ANOVA, or interaction effects by 2-factor ANOVA, differences between groups were analyzed by Dunnett’s post-hoc test comparing experimental groups to the non-irradiated (sham) controls, or all pairs Tukey-Kramer (software JMP Version 9.02, SAS Institute Inc). All data are presented as mean and standard deviations.

## Additional Information

**How to cite this article**: Schreurs, A.-S. *et al.* Dried plum diet protects from bone loss caused by ionizing radiation. *Sci. Rep.* **6**, 21343; doi: 10.1038/srep21343 (2016).

## Figures and Tables

**Table 1:** Average body weights and food consumption.

Diet/Tx	IR	Avg. Weight (g)	Avg. Food eaten(g/day)
CD1	−	27.1 + 2.3	NA
CD1	+	27.1 + 1.4	NA
CD1+DHLA	+	27.9 + 2.2	NA
CD1+IBU	+	27.6 + 2.0	NA
CD3	−	25.9 + 1.4	2.8 + 0.1
CD3	+	25.2 + 1.4	2.9 + 0.2
CD3+DP	−	26.8 + 0.7	2.8 + 0.3
CD3+DP	+	25.3 + 0.8	3.0 + 0.2
CD2	−	26.9 + 1.1	2.8 + 0.3
CD2	+	26.6 + 0.9	2.9 + 0.3
CD2+AOX	−	27.6 + 1.2	2.7 + 0.2
CD2+AOX	+	26.6 + 1.8	2.8 + 0.2

Average body weights at the end of the study (mean + SD). Food intake was measured over a 10-day period, and averaged to assess daily food consumption. NA = Not Available.

**Table 2:** Cortical bone structure.

Cortical bone parameter	CD3 + Sham	CD3 + IR	DP + Sham	DP + IR	p value
Bone Area (mm^2^)	0.766 + 0.0750	0.731 + 0.0351	0.763 + 0.0612	0.764 + 0.0542	n.s.
Bone Perimeter (mm)	7.06 + 0.438	6.91 + 0.208	7.07 + 0.346	7.05 + 0.240	n.s.
Cortical Thickness (mm)	0.260 + 0.0141	0.252 + 0.00745	0.260 + 0.0155	0.260 + 0.0133	n.s.
Mean Polar Moment of Inertia (mm^4^)	0.222 + 0.0454	0.201 + 0.0192	0.220 + 0.0374	0.220 + 0.0301	n.s.
Tissue Mineral Density (mg/cm^3^)	929 + 3.67	923 + 11.7	926 + 10.8	926 + 7.74	n.s.

Features of cortical bone that contribute to whole bone mechanical properties were measured by microcomputed tomography (mean + SD). TMD = Tissue Mineral Density. n.s. = not significant.
